# Allele-Specific Network Reveals Combinatorial Interaction That Transcends Small Effects in Psoriasis GWAS

**DOI:** 10.1371/journal.pcbi.1003766

**Published:** 2014-09-18

**Authors:** Sharlee Climer, Alan R. Templeton, Weixiong Zhang

**Affiliations:** 1 Department of Computer Science and Engineering, Washington University, St. Louis, Missouri, United States of America; 2 Department of Biology, Washington University, St. Louis, Missouri, United States of America; 3 Department of Genetics, Washington University, St. Louis, Missouri, United States of America; 4 Institute of Evolution, and Department of Evolutionary and Environmental Biology, University of Haifa, Haifa, Israel; 5 Institute for Systems Biology, Jianghan University, Wuhan, Hubei, China; The University of North Carolina at Chapel Hill, United States of America

## Abstract

Hundreds of genetic markers have shown associations with various complex diseases, yet the “missing heritability” remains alarmingly elusive. Combinatorial interactions may account for a substantial portion of this missing heritability, but their discoveries have been impeded by computational complexity and genetic heterogeneity. We present BlocBuster, a novel systems-level approach that efficiently constructs genome-wide, allele-specific networks that accurately segregate homogenous combinations of genetic factors, tests the associations of these combinations with the given phenotype, and rigorously validates the results using a series of unbiased validation methods. BlocBuster employs a correlation measure that is customized for single nucleotide polymorphisms and returns a multi-faceted collection of values that captures genetic heterogeneity. We applied BlocBuster to analyze psoriasis, discovering a combinatorial pattern with an odds ratio of 3.64 and Bonferroni-corrected p-value of 5.01×10^−16^. This pattern was replicated in independent data, reflecting robustness of the method. In addition to improving prediction of disease susceptibility and broadening our understanding of the pathogenesis underlying psoriasis, these results demonstrate BlocBuster's potential for discovering combinatorial genetic associations within heterogeneous genome-wide data, thereby transcending the limiting “small effects” produced by individual markers examined in isolation.

## Author Summary

Most complex diseases arise due to combinations of genetic factors, yet current genome-wide association studies (GWAS) typically examine individual genetic markers in isolation because of the complexity of considering a prohibitively large number of marker combinations. Another complication for GWAS stems from *genetic heterogeneity*, in which different subsets of individuals develop a given disease due to different sets of genetic factors. We present BlocBuster, a network-based method that addresses these challenges and extracts inter-correlated genetic markers that manifest significant associations with complex diseases. Our analysis of psoriasis GWAS data revealed a significant combinatorial genetic pattern, which was validated using stringent computational tests and replication in independent data. This pattern is more significant than other previously identified markers. We also compared Pearson's correlation coefficient and observed that it introduced more type I errors and produced a less structured network than BlocBuster; the former also broke the combinatorial pattern into pieces. In addition to improving prediction of disease susceptibility and broadening our understanding of the pathogenesis underlying psoriasis, these results demonstrate BlocBuster's effectiveness for discovering combinatorial genetic associations within heterogeneous backgrounds, thereby transcending the limiting “small effects” produced by individual markers examined in isolation.

## Introduction

Psoriasis is an incurable complex disease that is characterized by hyperproliferation and aberrant differentiation of the epidermis, coupled with marked cutaneous inflammation. Environmental triggers for onset of symptoms have been observed, yet genetic predisposition is strong and heritability has been estimated at 80% [1]. This disease appears with dramatic variations between populations. It is essentially nonexistent in Eskimo and South American Indian populations, affects 2%–3% of individuals with European ancestry [2], and has been reported as high as 11.8% in Kazach'ye, Russia [3].

The strongest known genetic risk factor for psoriasis, *PSORS1*, is within the major histocompatibility complex (MHC) region on chromosome 6 [4]–[7], a region that has been a primary focus of psoriatic research spanning at least 40 years [5], [8]–[10]. The area of interest includes *HLA-C*, *PSORS1C1* (aka *SEEK1*), *PSORS1C2* (aka *SPR1*), *PSORS1C3*, *CDSN*, and several additional genes and pseudogenes in a ∼300-kb region of 6p21.3. A number of genome-wide association studies (GWAS) focused on psoriasis have identified associated single nucleotide polymorphisms (SNPs) in this region, as well as other regions of the genome, e.g., those reported in [2], [11]–[16]. Table 1 lists a collection of SNPs with odds ratios of at least 1.4.

**Table 1:** Selection of previously identified risk alleles for psoriasis.

ID	Chr.	Position	Risk allele	Cases risk allele freq.	Controls risk allele freq.	OR	Closest gene
rs4406273	6	31266090	A	0.259	0.092	3.45	*WASF5P*
rs34536443	19	10463118	G	0.974	0.953	1.85	*TYK2*
rs2233278	5	150467189	C	0.090	0.058	1.61	*TNIP1*
rs9988642	1	67726104	T	0.952	0.929	1.52	*IL23R*
rs33980500	6	111913262	T	0.108	0.074	1.52	*TRAF3IP2*
rs12188300	5	158829527	T	0.132	0.095	1.45	*IL12B*

Shown are the SNP IDs, chromosome, position, risk allele frequencies [14], odds ratio (OR), and closest gene for all markers cited by Tsoi et al. [14] with an OR of at least 1.4. Positions follow Genome Build 37.3, as given by NCBI's dbSNP website (http://www.ncbi.nlm.nih.gov/projects/SNP/).

Overall, it is estimated that less than 20% of genetic heritability can be accounted for by previous discoveries [4], [17]. Furthermore, many of these loci have not been replicated using independent data, suggesting the existence of genetic heterogeneity in the pathogenesis of this disease, as was implicated in the early work of Burch and Rowell [18]. Furthermore, there are substantial uncertainties about the precise contributing mutations that are captured by linkage disequilibrium (LD) within each of the associated genomic regions [2]. Importantly, previous research has indicated that psoriasis arises due to the interactions of multiple genetic factors [19]; thereby further complicating efforts to understand this complex disease.

Identifying genetic risk factors and understanding the genetic basis of complex diseases, such as psoriasis, are central goals of medicine and biology. While GWAS have identified hundreds of individual genetic markers associated with complex diseases, it is clear that a substantial portion of heritability remains unexplained for the vast majority of these enigmatic phenotypes [20]. The susceptibility of complex diseases may be influenced by a multiplicity of genetic factors, as well as environmental factors. When considered individually, many contributing genetic factors may have a small or undetectable effect on the disease, making them difficult to identify [21], [22]. Moreover, the effects of these risk factors might not be simply additive; they may be compounded epistatically through sophisticated interactions [23], [24]. Standard GWAS only identify single variants associated with the phenotype of interest as they lack power to detect epistasis [25]. An additional impediment to identification of combinatorial genetic interactions is that the correction for multiple testing would be prohibitively enormous as the number of tests grows exponentially in the number of markers considered simultaneously [25]. In fact, conducting such tests quickly becomes intractable. When the number of markers is in the hundreds of thousands, or millions, even just examining every pair of two markers can be computationally demanding. For example, one million markers can be paired in 499,999,500,000 unique ways. The computational challenge increases exponentially for higher-ordered combinations – examining every combination of three markers requires examining 1.7×10^17^ trios. Consequently, directly testing every trio or higher-ordered combination is computationally intractable using currently available resources and will likely remain infeasible for the foreseeable future [26].

In order to examine pair-wise epistasis involving two SNPs, several methods have been introduced in which subsets of SNPs are selected using previously known biological information, and pair-wise interactions are computed over these subsets [27]–[29]. For example, Strange et al. tested pair-wise interactions for a set of SNPs that had each shown associations with psoriasis when tested independently in standard GWAS, and discovered an interaction between *HLA-C* and *ERAP* [30]. Chen et al. later tested ten SNPs that had been indicted to be associated with psoriasis, but no significant epistasis between pairs of SNPs was found [4]. Analyses examining subsets of SNPs reduce the computational burden but limit discoveries to these subset selections. On the other hand, methods such as PLINK's Fast Epistasis [31] and a statistic introduced by Wu et al. [32], [33] are computationally efficient and can be used to blindly test all pairs of SNPs. However, these trials impose hefty multiple testing corrections, resulting with little progress in this area.

Alternatively, haplotypes have the potential to provide more power than single SNPs [34]–[37]. A haplotype is a set of contiguous allele-specific markers that ideally span a “haplotype block” in which high LD is exhibited. Haplotype data are not directly acquired for GWAS as current profiling methods provide genotypes that specify the two alleles for a given SNP, but are unable to align the alleles for each of the homologous chromosomes. Computational haplotype inference methods are commonly employed to phase genotypes into two homologous haplotypes, each of which possesses a set of contiguous SNP alleles. However, most computational approaches phase all SNPs supplied within each region, which may result with the inclusion of un-informative or even misleading markers.

The *HLA-Cw6* haplotype family consisting of *HLA-Cw0602—Cw0613* alleles within *PSORS1* has shown strong association with psoriasis [5], [19]. This haplotype is defined by the CCATCCG SNP alleles at positions 213, 218, 341, 361, 387, 459, and 540 of NM_002117.4 [5]. Nair et al. [5] completely sequenced the *PSORS1* region for one psoriatic individual and four controls, genotyped risk alleles in 678 psoriatic families, and employed three computational haplotype reconstruction methods as well as a combinatorial analysis to implicate the *HLA-Cw6* haplotype as the most probable source of susceptibility within the *PSORS1* locus for early-onset psoriasis of individuals with European ancestry. It should be noted that their analysis focused on age of onset and it only included exons from known protein-coding genes. Non-coding regions were left unexamined.

It has been demonstrated that complex phenotypes, e.g. human height [38], may be associated with markers that are not necessarily in close proximity but rather even span across the genome. Therefore, it is desirable to explore general combinations of alleles without imposing restrictions regarding genomic proximity. A number of approaches have been proposed for identifying patterns of multiple markers that may be linked or unlinked, yet interact to contribute to a phenotype of interest. For example, Chen et al. [4] conducted combinatorial analyses of ten SNPs that had been previously identified to each have individual associations with psoriasis. These ten SNPs have been highly replicated and included a SNP tagging *HLA-Cw6*. They tested risk predictions via simple allele counts of multiple markers as well as weighted combinations and observed that the contribution due to the *HLA-Cw6* SNP was about equal to the contribution of the other nine SNPs combined. The authors noted the possibility of overfitting in their weighted approach due to the use of the same data for model construction and subsequent analysis [4]. More generally, the limitation to SNPs with previously-identified associations eradicates the possibility of identifying interacting factors that exhibit negligible associations when examined in isolation.

One approach to address this limitation is to consider all genome-wide SNP states together for each individual and apply a regression analysis or maximum likelihood estimate over the genomic similarities between individuals. Yang et al. used this approach to estimate variance in human height [39], a highly heritable trait that is associated with hundreds of genetic variants [40]. This type of approach is advantageous as it dramatically reduces the burden of multiple testing corrections, with potential to reduce false-negative signals. On the other hand, for many complex traits the number of causal loci associated with a phenotype of interest may account for a small or negligible proportion of variation between individuals. For example, two individuals might exhibit high genomic similarity due to shared ancestry dominating the similarity measure; while another two individuals with less common ancestry, yet sharing a handful of causal alleles contributing to a given phenotype, might exhibit low genomic similarity. This problem can be exacerbated by genetic heterogeneity, as described below. In short, the abstraction of many hundreds of thousands of variable markers into a similarity measure that captures a small handful of markers associated with a particular phenotype could be expected to require extremely large sample size or the use of previously known information.

Several network construction approaches utilizing known information have been introduced to capture combinations of markers with phenotypic associations, such as nested clade analysis [41], treescanning [42], simulated evaporative cooling networks [43], SNPrank [44], Hua et al.'s SNP-SNP networks [28], statistical epistasis networks [45], and Li et al.'s two-step method [27]. These methods utilize phenotypic information and/or biological knowledge in construction of networks that are subsequently explored. Networks that have strong community structure can be partitioned into clusters, or *communities*, of nodes such that there is a high density of edges within each community and few edges spanning between communities. Many approaches utilize clustering methods to partition the network, followed by the use of reference databases – e.g. Gene Ontology [46], KEGG [47], DAVID [48], or MetaCore – to evaluate gene enrichment for each community. Due to the assumptions and/or biases that are introduced during network construction, it is difficult to accurately evaluate the contributions using enrichment analyses based upon reference databases.

**Figure 1:**
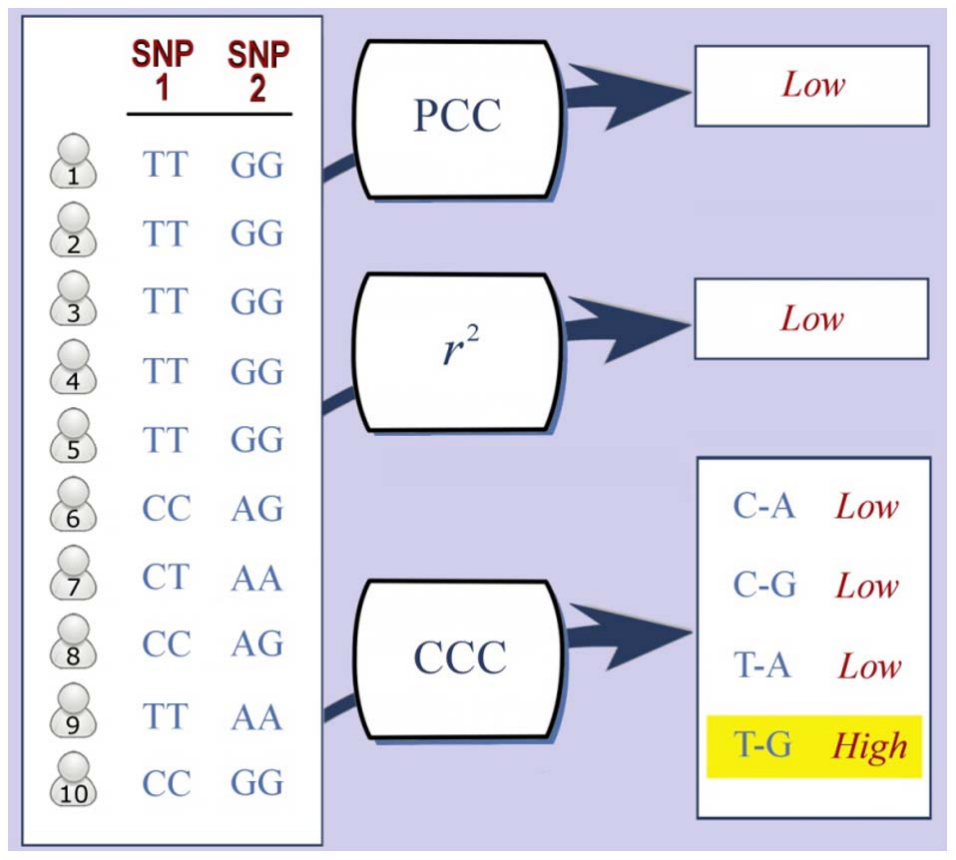
Genotypes for ten individuals for a pair of SNPs. The first five individuals are perfectly correlated, but the others are not correlated at all. The absolute value of PCC is 0.3 and *r* ^2^ returns 0.0, due to the uncorrelated individuals. CCC supplies four correlation values, each of which corresponds to a specific type of correlation. These values are low for three of the possible combinations, but a high value of 0.7 for the T-G combination was returned.

In addition to the challenges of combinatorial interactions, another serious issue that is impeding identification of phenotypic associations in GWAS is that most complex diseases are subject to genetic heterogeneity, in which different groups of individuals develop the same disease due to different genetic factors or gene-by-environment interactions. Heterogeneity can manifest as different mutations within a single gene or as mutations within different genes. For example, cystic fibrosis can arise due to more than 1,000 different mutations within a single gene, *CFTR* [49], and retinitis pigmentosa can arise due to specific mutations in any one of at least 45 different genes [50]. The inability to replicate psoriatic associations in populations that are distinct from the original population may be due to heterogeneity [51]. Critically, heterogeneity has not been adequately quantified nor even captured by the currently available measures of correlation, which is one of the most fundamental concepts in statistical analyses of genetic data. Two commonly adopted correlation measures in the biomedical domain are Pearson's correlation coefficient (PCC) [52] and the linkage disequilibrium measure, *r* ^2^. Importantly, each of these popular correlation methods returns a single scalar that can be crippled by heterogeneity. In fact, all of the correlation measures that we have examined, including PCC and *r* ^2^, as well as dot product and entropy, are global, in that individuals in the entire group are viewed as a whole, and thus subtle but critical subgroup structures, which manifest heterogeneity of the individuals in the group, are ignored. Figure 1 illustrates a simple example for two SNPs, where half of the individuals are perfectly correlated, while the other individuals are not correlated at all. PCC and *r* ^2^ have low values, due to penalization for the uncorrelated individuals. In short, current correlation measures treat the correlation for all of the individuals as a whole, and consequently, fail to accommodate genetic heterogeneity within the sample studied.

In order to address these pressing challenges, we present BlocBuster, a systems-level, allele oriented network strategy designed to generate viable biological hypotheses of epistatic and/or additive interactions of genetic variations with a holistic and unbiased approach that honors genetic heterogeneity. BlocBuster has two key distinctions from the previous approaches. First, we introduce a significant extension to the custom correlation coefficient (CCC, “triple C”) [53], a metric designed to capture genetic heterogeneity by computing a multi-faceted collection of correlation measurements. Each facet independently captures a single homogeneous type of correlation. Second, we build an unbiased network of SNP alleles, identify clusters of correlated alleles within the network, test each entire cluster for phenotypic association, and rigorously validate the results. During network construction, we consider all individuals together, without phenotype labeling, and consider all markers simultaneously, thereby assessing relationships *in toto*. In our current implementation, each bi-allelic SNP is represented by two nodes, one for each SNP allele, which can be readily extended to multi-allelic SNPs. CCC is computed for every pair of SNPs, thereby revealing correlations that arise regardless of genomic distances between the SNPs. Significant values within the CCC vectors are represented by edges between the nodes representing the correlated alleles. After the network is built, we retrieve clusters of inter-correlated nodes that arise naturally separated from each other and do not require partitioning. Each of the corresponding patterns of SNP alleles is then tested for variation between phenotypic groups. Consequently, BlocBuster extracts patterns of inter-correlated haploid markers, referred to as *blocs*. These blocs seamlessly capture genetic heterogeneity as they are built upon the multi-faceted CCC metric that treats each pair of SNP alleles independently, thereby avoiding reduction of the correlation value by subgroups of individuals that lack correlation for the pair of alleles. An allele-specific network is utilized to eliminate the merging of both SNP allele states into a single node, which can lead to false positive signals, as described below. Our approach is direct and efficient, and scales efficiently to millions of markers with reasonable computational resources.

In order to ensure accuracy of results, BlocBuster employs a series of computational validation trials, including two types of permutation tests, bootstrapping trials, variations of network density, and visual inspection. These computations economically screen the results and can be utilized prior to investment in replication trials using independent data.

Aiming at identification of combinatorial interactions of SNP alleles underlying pathogenesis of psoriasis, we applied BlocBuster to genome-wide data for psoriatic cases and normal controls. This analysis identified a bloc of SNP alleles that is significantly associated with psoriasis and improves upon previous results by supplying a precise allelic pattern within the major histocompatibility complex (MHC). This newly identified genetic pattern was rigorously validated using multiple computational screening tests and was subsequently replicated in independent data, thereby ensuring its accuracy and suitability for further research efforts. Finally, we compare and contrast our approach with Pearson's Correlation Coefficient (PCC) and observe that the PCC network had substantially weaker community structure, was more likely to introduce false-positive correlations, and required three times as much computation time.

## Results

In order to discover combinatorial interactions in heterogeneous samples of a given complex disease, we developed a novel computational approach, referred to as BlocBuster, which identifies clusters, or blocs, of correlated SNP alleles and subsequently tests these blocs for phenotypic associations. Briefly, an allele-specific network is constructed in which each SNP allele is represented by a node and edges are placed between pairs of nodes representing SNP alleles that exhibit significant pair-wise correlations. In order to address genetic heterogeneity, we utilize a multi-faceted correlation metric that is customized for SNP data, referred to as CCC. Note that the CCC computation is conducted for the entire sample of *all* individuals and the network construction is blind to phenotype status. Furthermore, CCC is computed for every pair of SNPs, providing a holistic systems-level network. After the network is constructed, groups of nodes that are connected by edges are easily identified as they are completely isolated from each other. Then the entire pattern of SNP alleles represented by each bloc is tested as a whole for association with the phenotype. These patterns are comprised of specific SNP alleles and can be considered as a type of haplotype – with two noteworthy exceptions: (1) only SNP alleles that exhibit inter-correlations are included and (2) the SNPs are not necessarily contiguous and are included regardless of genomic position.

In this section, we present the results provided by BlocBuster for psoriasis GWAS data. These results were carefully validated using a series of computational trials and these outcomes are overviewed. The most significant result is a bloc of 17 SNP alleles that is strongly associated with psoriasis. We replicated this result by utilizing independent data and found that the bloc has a stronger association in the replication data than in the discovery data. Finally, we compare CCC with a standard correlation metric, Pearson's correlation coefficient (PCC), and observe the benefits of utilizing a metric that is customized specifically for SNP data and is designed to accommodate heterogeneity.

### Network analysis of psoriasis

We used both psoriatic cases and normal controls in the GAIN General Research Use (GRU) genome-wide data to construct the BlocBuster network for this complex disease. These data consisted of 443,020 autosomal SNPs for 929 cases and 681 controls (see Methods). The correlation between every pair of SNPs was computed using CCC. We set the number of edges in the network equal to the number of SNPs, consequently selecting edges with the highest 443,020 CCC values – see Methods for discussion of this parameter selection and validation trial results for significance of this threshold and sensitivity of its value. The network was comprised of 886,040 nodes as each SNP allele was represented by a node. Consequently, the average degree of each node in the network was one. If the edges were uniformly distributed, the network would consist of 443,020 *doubletons*, each of which was comprised of a single edge connecting two nodes; with every node connected to precisely one other node in the network.

In sharp contrast to a network with uniform distribution, the observed network exhibited strong community structure. Instead of doubletons spread across the network, there were a large percentage of *singleton* nodes with no incident edges, and many discrete blocs of densely connected nodes, with each bloc isolated from one another. Specifically, 631,462 (71.3%) of the nodes were singletons, and there were 54,425 discrete blocs, ranging from 2 to 313 nodes, with an average of 4.7 nodes per bloc.

Importantly, the blocs arose naturally separated in the network and there was no need to employ methods such as clustering strategies to partition the nodes. For each of the 54,425 blocs, the frequencies of the entire corresponding SNP allele pattern were tallied and the odds ratio with 95% confidence interval (CI) and Bonferroni-corrected p-value, based on the G-test of independence, were computed between cases and controls (see Methods). Any individual that was missing more than 5% of the genotypes in the bloc was not included in these calculations. Note that other than these missing genotypes, the *entire* SNP allele pattern must be present to be counted in the bloc frequency. This policy assumes that the entire pattern is required for the phenotypic association for a given subset of individuals. However, it is possible that one or more of the SNP alleles in the bloc is not essential. The visual validation described below facilitates the observation of this situation, should it arise.

**Figure 2:**
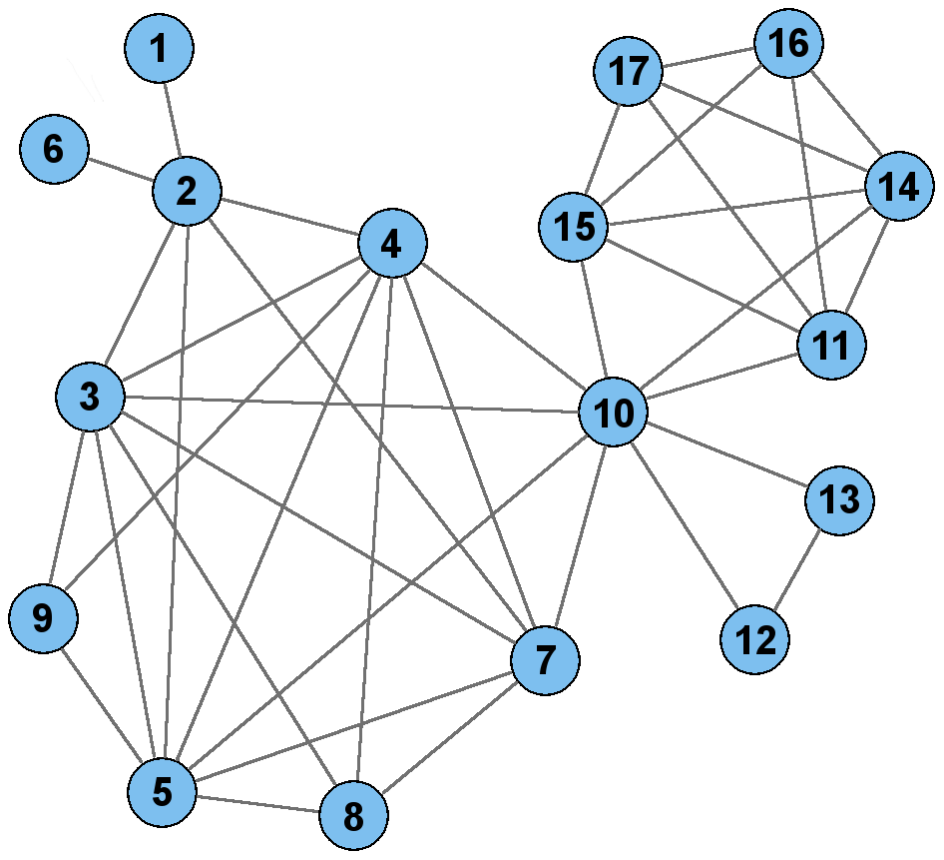
Plot of 17-node bloc, *ps_1*. Each node represents a SNP allele and each edge represents a significant correlation between the SNP alleles representing its endpoints. The pattern corresponding to this bloc exhibited a risk association with psoriasis, with an odds ratio of 3.64 (CI: 2.75–4.80) and Bonferroni-corrected p-value of 5.01×10^−16^ in the discovery data and odds ratio of 3.86 (CI: 2.98–5.01) and Bonferroni-corrected p-value of 1.81×10^−25^ in the validation data.

This analysis revealed a single bloc, referred to as *ps_1*, which was comprised of 17 SNP alleles and had a significant odds ratio and CI (Figure 2). Individuals missing more than 5% data for these 17 SNPs were omitted, leaving sample sizes of 785 cases and 585 controls. This pattern had a “protective” association with frequencies of 0.179 and 0.265 for the psoriatic cases and controls, respectively, with an odds ratio of 0.605 (CI: 0.482–0.759). However, the p-value was not significant after Bonferroni correction. On the other hand, the alternate alleles for all 17 SNPs comprised a risk pattern that had frequencies of 0.220 and 0.072 for cases and controls, respectively. This risk pattern had an odds ratio of 3.64 (CI: 2.75–4.80) and Bonferroni-corrected p-value of 5.01×10^−16^. The risk pattern is more significant than the protective pattern when comparing cases to controls. However, when considering all individuals together, the protective pattern is more pronounced (with a frequency of 0.216) than the risk pattern (with a frequency of 0.157), which is likely the reason that the protective pattern appeared in the network that was constructed using all individuals without phenotypic labeling.

Table 2 lists the 17 nodes in *ps_1*, along with alleles and their individual frequencies. The SNPs within *ps_1* span ∼211 kb, from positions 31054511 to 31265057 in the MHC on chromosome 6. Two SNPs are located within known genes: rs3130573 in *PSORS1C1* (aka *SEEK1*) and *PSORS1C2 (aka SPR1)*, and rs1265078 in *CCHCR1* (coiled-coil α-helical rod protein 1, aka *HCR*). All three of these genes have been previously associated with psoriasis [54], [55].

**Table 2:** Description of SNP alleles corresponding to the nodes in *ps_1*.

*Node #*	*Risk Allele*	*Freq. Cases*	*Freq. Controls*	*OR*	*rsID*	*Chr. Position*
1	G	0.431	0.324	1.58	rs3130573	31106268
2	C	0.421	0.300	1.70	rs1265078	31112602
3	T	0.394	0.266	1.79	rs3130467	31187075
4	C	0.391	0.260	1.83	rs3130517	31190303
5	T	0.381	0.252	1.83	rs3130713	31205617
6	T	0.530	0.438	1.45	rs3130685	31206206
7	C	0.360	0.233	1.85	rs2394895	31206979
8	A	0.469	0.346	1.67	rs3130955	31054511
9	A	0.516	0.413	1.52	rs9263967	31186245
10	T	0.404	0.256	1.97	rs2844627	31229462
11	T	0.298	0.150	2.41	rs12191877	31252925
12	C	0.513	0.401	1.57	rs2524163	31259579
13	A	0.513	0.405	1.55	rs2243868	31261276
14	C	0.341	0.208	1.97	rs2894207	31263751
15	A	0.296	0.154	2.31	rs9468933	31265057
16	G	0.424	0.288	1.82	rs7773175	31240959
17	A	0.404	0.291	1.65	rs9380237	31264392

All SNPs are located on chromosome 6. The node numbers correspond to the numbers in Figures 2 and 4. Shown are the frequencies of the risk alleles for psoriatic cases and controls, odds ratio (OR) for each individual SNP, SNP IDs, and chromosomal positions, where rsID is the dbSNP assigned reference SNP identification number and positions are for Genome Build 37.3, as given by NCBI's dbSNP website (http://www.ncbi.nlm.nih.gov/projects/SNP/).

### Computational validations

In order to evaluate the robustness of our results, we ran five computational validations: two types of permutation tests, trials in which we varied the network density, bootstrapping trials, plus visualization of results. The first series of permutation tests were conducted to determine a significant G-test score given multiple tests (see Methods). In these trials, the phenotypic labels of individuals were permuted prior to computing G-test scores for each of the 54,425 blocs. Each permutation trial destroys the associations between genotypes and the phenotype, but maintains the statistical properties of the whole so that they can be used as background for this significance analysis. These trials indicated a G-test score of 23.7 corresponds to a corrected p-value of 0.05. A G-test score of 24.1 corresponds to the same significance when using Bonferroni correction, indicating these two approaches for multiple testing corrections are similar for this study.

The second series of permutation tests were used to remove inherent correlations among SNP alleles in the data in order to verify that it is unlikely that type I errors were introduced during computations of correlations. For each SNP, we randomly shuffled the genotypes across all individuals. This randomization breaks inherent correlations while each SNP retains the same allele frequencies and balance of genotype states as in the original data. Consequently, it is not expected that there would be significant correlations within the permuted data. These trials aim to estimate the highest CCC value that might arise by random chance for uncorrelated data drawn from these samples. The maximum CCC value for 9.8×10^10^ pairs of SNPs with permuted genotypes was 0.6515. The lowest CCC value representing an edge in the original network was 0.6949. This result indicates that it is not likely that there were any edges representing false-positive correlations in the original network.

In the third validation trial, we varied the density of the network to test the sensitivity to this parameter. For a given number of edges *n*, the highest *n* CCC values were determined and the corresponding SNP allele pairs were connected by edges in the network. We constructed networks with 50,000 to 500,000 edges, in increments of 50,000, and tracked *ps_1* within these networks. The CCC thresholds for edge placement for these trials varied from 0.7501 (for 50,000 edges) to 0.6906 (for 500,000 edges), which is substantially higher than the maximum CCC value of 0.6515 that was produced during the permutation trials, indicating that it is not likely that false positives arose during these trials.

The sparse networks with no more than 300,000 edges did not include *ps_1*. The 350,000 edge network possessed a bloc with 16 of the 17 nodes in *ps_1*; node 6 was not included in this bloc. The association of this bloc was similar to the original 17-node bloc as it had frequencies of 0.218 and 0.071 for cases and controls, respectively, with an odds ratio of 3.64 (CI: 2.83–4.67) and Bonferroni corrected p-value of 1.81×10^−23^. The 400,000 edge network possessed the entire 17-node bloc. The 450,000 edge network added another node to the bloc, rs2442736. This bloc had frequencies of 0.137 and 0.039 for the cases and controls, respectively, with an odds ratio of 3.91 (CI: 2.81–5.45) and Bonferroni-corrected p-value of 1.10×10^−14^. For the 500,000 edge network, the bloc grew to 30 nodes, had frequencies of 0.020 and 0.006, and an odds ratio of 3.32 (CI:1.54–7.19). The p-value was not significant after correcting for multiple testing. Table S1 lists these 30 SNP alleles. Overall, *ps_1* was significant for a range of network densities. However, when the network grew to a half million edges, this bloc grew substantially, its frequency fell to less than two percent, and it exhibited a weaker association with the phenotype.

In our fourth validation, we tested the sensitivity of the selection of individuals with a series of bootstrapping trials. This resampling technique evaluates the stability of results computed over a sample drawn from a population and has been shown to be more accurate than methods that are based on asymptotic approximation or normality assumptions [56]. We conducted 1,000 bootstrapping trials in which we randomly selected half of the cases and half of the controls and computed the odds ratios and 95% confidence interval for *ps_1*. Over the 1,000 computations, the odds ratios had a mean of 3.66 with CI: 3.64–3.69. This result is slightly better than the results found for the entire dataset, and the confidence interval is tighter, as it was based on 1,000 trials. The average p-value was 2.91×10^−11^, which is larger than the original result, likely due to the fact that these trials each had half of the original sample size. Overall, these results indicate robustness to sample selection as randomly selected subsets of the individuals yield strong phenotypic associations.

**Figure 3:**
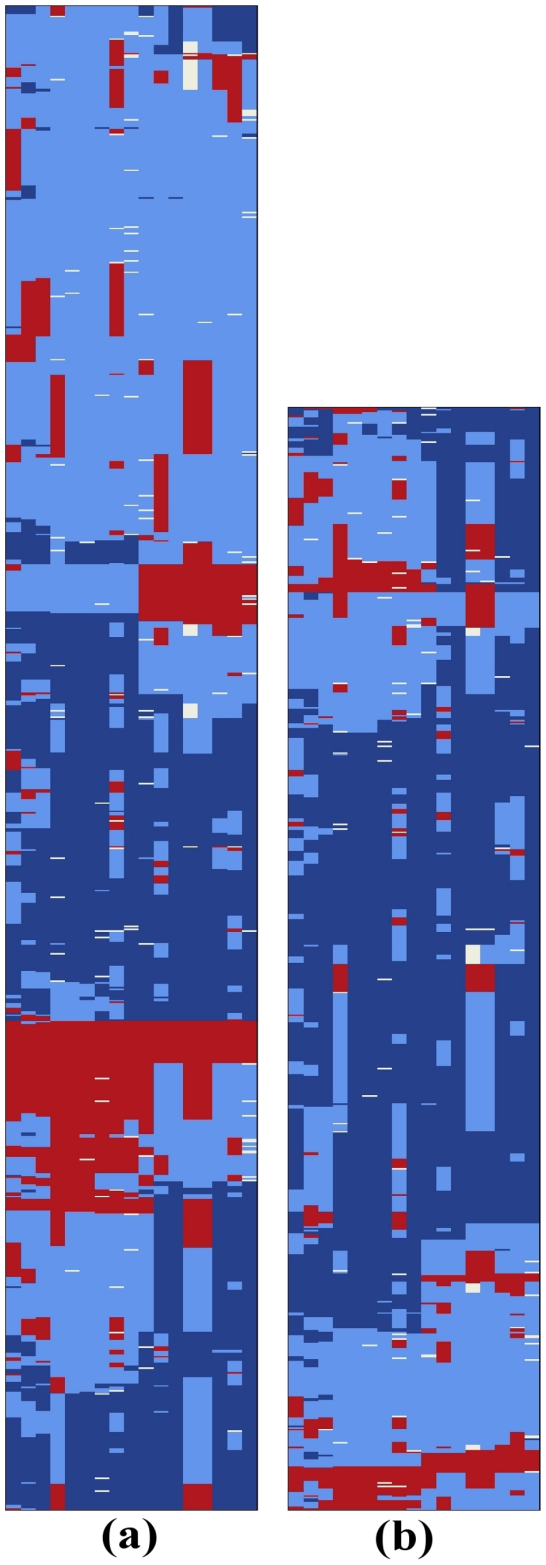
Genotype states of (a) 929 psoriatic cases and (b) 681 controls for bloc *ps_1*. (Best viewed in color.) Each row represents an individual and each column represents a SNP. Dark blue represents a homozygote for the protective allele, light blue represents a heterozygote, red represents a homozygote for the risk allele, and white indicates missing data. Individuals (rows) were rearranged using TSP+*k* [77] in order to place similar individuals near each other and enhance visualization of patterns.

Finally, we extracted the genotypes for the SNPs corresponding to *ps_1*, and plotted them for visual inspection, as shown in Figure 3. These plots illustrate the variation of the genotypes across psoriatic cases and controls.

### Replication in independent data

We tested the identified bloc *ps_1* using the GAIN Autoimmune Disease Only (ADO) data. These data consisted of 443,020 genotype states for 439 psoriatic cases and 728 controls. For these data, the frequencies of the entire *ps_1* bloc were 0.260 and 0.083, with an odds ratio of 3.86 (CI: 2.98–5.01) and p-value of 1.81×10^−25^. Note that only one bloc was tested, so there was no correction for multiple testing. These results are even stronger than those for the discovery data, demonstrating the validity of the association of this bloc with psoriasis.

### Comparison between CCC and PCC

In our analysis, we found that the linkage disequilibrium measure, *r* ^2^, required many thousands times more computation time than CCC or PCC, and is not practical for studies involving large numbers of SNPs. Accordingly, in this section we disregard *r* ^2^ and focus on comparisons between PCC with CCC. Due to computational demands, we used the 30,178 SNPs from chromosome 6 for the following trials. We first computed all pair-wise correlations using both metrics and compared the network structures that each produced. Second, we ran permutation trials and compared the numbers of false positives produced by each method, as well as average computation time. Third, we extracted the clusters in the PCC network that possessed the SNPs in *ps_1* and compared differences in the results.

First, we created two networks for the SNPs using each of the correlation metrics. BlocBuster networks possess two nodes for each SNP thereby capturing allele-specific correlations. In comparison, PCC does not return the alleles associated with a correlation, so only one node was used to specify each SNP. In order to compare the two methods, we built a CCC network with only one node per SNP and placed an edge between a SNP pair if any of the four allele-specific CCC facets exceeded the threshold. Setting the number of edges equal to the number of SNPs for chromosome 6 corresponded to thresholds of 0.6990 and 0.7350 for CCC and PCC, respectively.

These two networks were compared for community structure. Strong community structure in a network opposes uniform edge distribution and is instead exemplified by clusters of tightly interconnected nodes such that few edges connect these clusters [57]. Overall, the CCC network had substantially stronger community structure than the PCC network as the edges in the latter were more dispersed throughout the network, as summarized in Table 3. In particular, the CCC network had only 934 doubletons while the PCC network possessed 2,550 of these dispersed edges. Furthermore, the CCC network possessed substantially higher percentages of nodes that were singletons than the PCC network (49.73% vs. 29.25%). Consequently, all of the edges in the CCC network were concentrated over about half of the network nodes, while the PCC edges were spread over about 70% of the nodes. Finally, the largest cluster in the CCC network contained 290 nodes with 2,400 edges, while the largest in the PCC network was comprised of 410 nodes and 2,402 edges. These clusters possessed almost exactly the same number of edges, yet the PCC cluster was comprised of substantially more nodes and was notably less dense. Overall, despite the fact that the networks possessed the same numbers of nodes and edges, the edges were more highly concentrated into fewer clusters in the CCC network and more dispersed in the PCC network.

**Table 3:** Comparisons between PCC and CCC.

% of Nodes that were Singletons		# of Doubletons		Average Cluster Size		Largest Cluster (# of nodes/# of edges)	
PCC	CCC	PCC	CCC	PCC	CCC	PCC	CCC
29.25%	49.73%	2,550	934	3.89	5.38	410/2,402	290/2,400

Networks were constructed for chromosome 6 using PCC and CCC to determine edge placements. The number of nodes and number of edges were the same for the PCC and CCC networks. Shown are the percentages of nodes that were singletons (nodes with no adjacent edges), number of doubletons (two nodes connected by an edge and isolated from other nodes), average number of nodes per cluster, and the number of nodes and edges in the largest cluster. Overall, the CCC network exhibited stronger community structure than the PCC network.

In our second comparison we ran ten permutation trials in which genotypes were randomly reordered over the individuals as previously described, computing CCC and PCC on each of the permuted datasets. The maximum PCC value for the entire networks ranged from 0.7068 to 1.0000 over the ten trials, with an average of 0.8534. Despite the fact that true correlations should have been eliminated from the data, there were a total of seven edges with PCC values of 1.0000. The maximum CCC values ranged from 0.6474 to 0.6492, with an average of 0.6483. These results suggest that CCC is less likely than PCC to produce spurious edges. It also indicates that the original PCC network might have possessed false positive edges as the threshold was 0.7350, while it is unlikely that the original CCC network possessed false positive edges with its threshold of 0.6990.

These trials also provided an opportunity to compare average computation time requirements. The CCC trials averaged 135 minutes and PCC averaged 402 minutes per trial; the former was three times faster than the latter.

**Figure 4:**
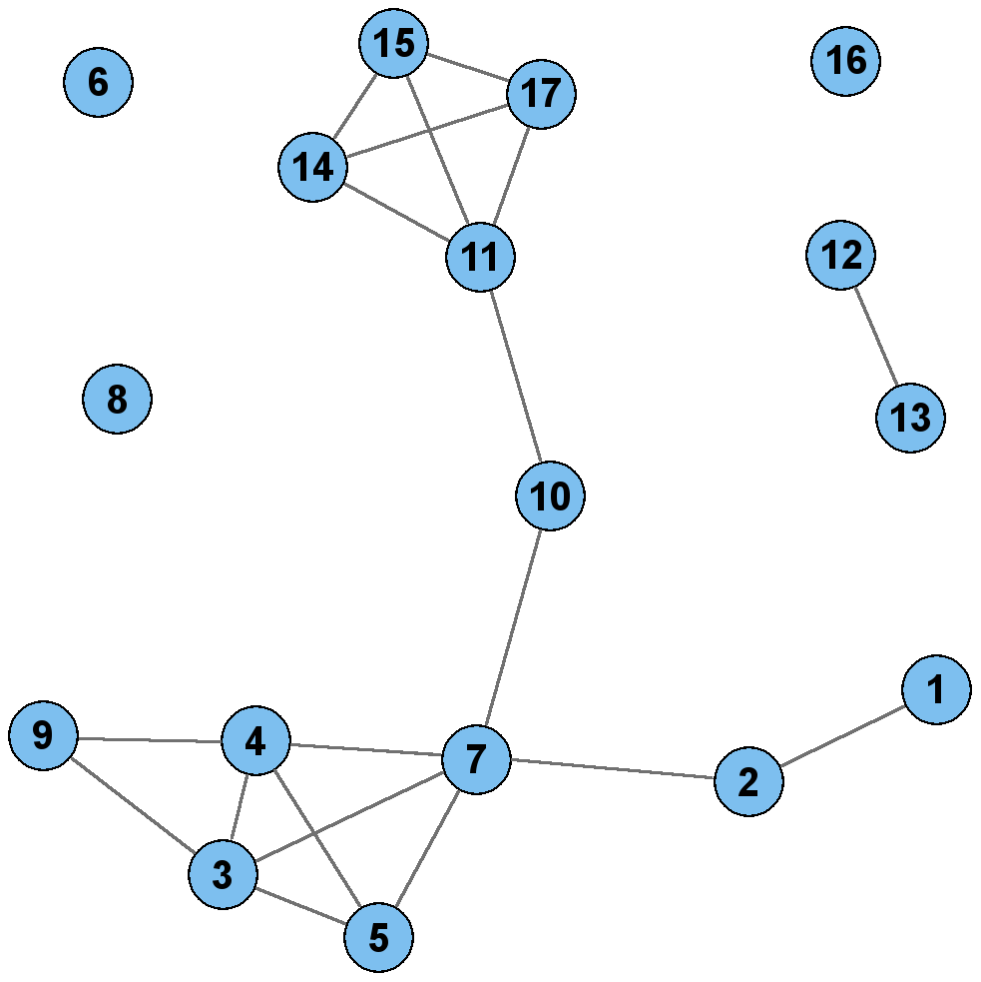
Plot of PCC edges for the 17 SNPs in the *ps_1* bloc. BlocBuster connected these 17 nodes into a single connected component, while PCC separated them into disconnected components. Overall, PCC identified 19 pair-wise correlations and CCC identified 39 correlations amongst these SNPs, as represented by edges in this plot and Figure 2.

Finally, the clusters in the PCC network possessing the SNPs in bloc *ps_1* were examined. Twelve of the 17 SNPs in *ps_1* comprised one cluster in the PCC network, two additional SNPs were connected together as a doubleton, and the other three were singletons, as shown in Figure 4. As shown in the figure, there were only 19 significant PCC correlations amongst these 17 SNPs, whereas there were 39 significant CCC correlations. This result demonstrates an example in which CCC identified a larger pattern with strong inter-correlations than those identified using PCC.

## Discussion

Psoriasis is a common complex disease that has been extensively studied and several GWAS have been previously conducted [2], [11]–[16]. Individual SNPs associated with psoriasis [14] with odds ratios as high as 3.45 have been identified (Table 1). By employing an unbiased, allele-specific network approach, BlocBuster identified a bloc, *ps_1*, consisting of a combination of 17 SNP alleles that are highly inter-correlated and exhibit a psoriatic risk association as a whole with an odds ratio of 3.64 (CI: 2.75–4.80) and Bonferroni-corrected p-value of 5.01×10^−16^ in the discovery data. This bloc had an odds ratio of 3.86 (CI: 2.98–5.01) and p-value of 1.81×10^−25^ in the validation data. BlocBuster's success in replicating a 17-SNP allele pattern in independent data reflects the robustness of the approach. In the network, each edge within the bloc represents a strong allele-specific correlation that is not weakened by genetic heterogeneity. As shown in Figure 2, the 17 SNP alleles are densely interconnected with high pairwise CCC correlations, while none exhibit such correlations for any other SNP allele outside the bloc. The use of a multi-faceted correlation metric and the retention of allele-specific information in network construction are key factors in BlocBuster's replication success.

**Figure 5:**
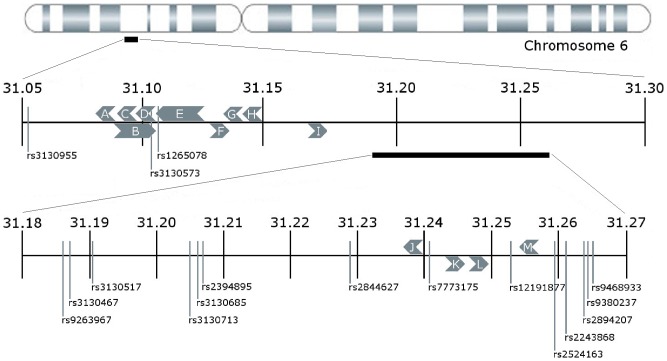
Map of the *ps_1* bloc on chromosome 6. Genes are represented by: (A) *C6orf15*, (B) *PSORS1C1*, (C) *CDSN*, (D) *PSORS1C2*, (E) *CCHCR1*, (F) *TCF19*, (G) *POU5F1*, (H) *PSORS1C3*, (I) *HCG27*, (J) *HLA_C*, (K) *USP8P1*, (L) *RPL3P2*, and (M) *WASF5P*. Two SNPs are within known genes: rs3130573 in *PSORS1C1* and *PSORS1C2*, and rs1265078 in *CCHCR1*. Shown are chromosomal positions (in Mb) according to Genome Build 37.3, as given by NCBI's dbSNP website (http://www.ncbi.nlm.nih.gov/projects/SNP/).

Node 1 of *ps_1* corresponds to SNP rs3130573, which is in the overlapping region of the *PSORS1C1* (aka *SEEK1*) and *PSORS1C2* (aka *SPR1*) genes. Both of these genes have been previously indicted to be associated with psoriasis [54]. Node 2 corresponds to rs1265078 in *CCHCR1* (coiled-coil α-helical rod protein 1, aka *HCR*). *CCHCR1* has been observed to have differential expression in psoriatic lesions when compared with either normal skin or eczema lesions [55]. It has been suggested that this gene is involved in the regulation of keratinocyte proliferation and may play a central role in the progression of psoriatic lesions [55]. The other SNPs in *ps_1* lie in intergenic regions as shown in Figure 5. Node 16 corresponds to rs7773175, which is upstream from *HLA-C*, a gene that has been associated with psoriasis over decades of research efforts [5], [15], [30], [58]–[60]. Node 11, rs12191877, was previously identified in a psoriasis GWAS conducted by Nair et al. [16]. In general, some of these SNP alleles might be directly involved in protein or regulatory variations underlying the pathogenesis of psoriasis and some might only hitch-hike along with causal variants. Further research is needed to discern these roles. Importantly, the results presented provide a highly defined pattern of SNP alleles to support such research.

The 17 SNPs in *ps_1* span a 211 kb region of chromosome 6, as shown in Figure 5. This region is within the Major Histocompatibility Complex (MHC) [61]. The MHC is a distinctive region of the genome and plays a fundamental role in human immune function. It has exceptionally high polymorphism [62] and possesses the greatest gene density in the genome [61]. Many MHC genes encode cell-surface antigens that mediate leukocyte interactions and they are associated with more than 100 human diseases, including common diseases such as diabetes, asthma, rheumatoid arthritis, as well as psoriasis, yielding strong selective pressures [63]. On the other hand, this region displays a heterozygote advantage by producing an enhanced immunocompetence; and diversity of these genes also increases fitness at the population level [64]. Several studies suggest there exist olfaction-mediated mate selection for increasing this diversity [65]–[70]. There exist hundreds of different alleles for the MHC genes [71] and each allele might appear on several different haplotype backgrounds as they may have arisen in various cycles of frequency fluctuations that arose during the balancing selection acting upon these genes throughout human history [62]. Indeed, the associated bloc lies within an exceptionally diverse genomic region and the occurrence of 17 SNP alleles in a single pattern with strong psoriatic association provides precise information for focusing future investigations.

Our network-based result is advantageous over the results from conventional GWAS. As shown in Table 2, the highest odds ratio for any single SNP in *ps_1* is 2.41 (SNP rs12191877). In contrast, the entire bloc as one unit exhibited odds ratios of 3.64 and 3.86 for the discovery and validation data, respectively. These results illuminate the discriminative power of combinatorial patterns and the capability of this approach for transcending single-marker methods. While the cardinality of the actual causal mutations which are tagged by the SNPs in *ps_1* may be less than 17 due to LD, recognition of multiple SNP alleles may reveal combinatorial interconnection among these SNPs underlying the etiology of psoriasis and tightens the fingerprint of the causal mutations, providing a fine-scaled pattern for future explorations of underlying genetic factors.

BlocBuster utilizes stringent validation procedures to efficiently weed out uninformative results to reduce resources that may have otherwise ensued in follow-up studies. These validation trials scrutinize blocs identified within the discovery data by examining their properties; specifically the robustness of the pattern and the improbability that it possesses type I errors or captures an incomplete and misleading portion of the data.

The permutation tests in which genotypes were randomly reordered indicated that it is highly unlikely an edge in the BlocBuster network appeared by random chance. On the other hand, PCC produced spurious edges and was not as capable to discern true correlations from noise, presumably due to its inherent inability of capturing genetic heterogeneity. Consequently, seven edges with PCC values of 1.0000 were computed from the permuted data in which correlations should have been eliminated.

In another validation, the genotype states of the identified blocs were plotted for visual inspection, as shown in Figure 3, to ensure the captured pattern is not misleading. The exceptional diversity characteristic of this region is apparent in the figure. However, note in these plots that the psoriatic cases have a smaller percentage of individuals that are mostly homozygous for the protective alleles (predominantly dark blue rows in Figure 3) and a substantially larger percentage of individuals that are mostly homozygous for the risk alleles (predominantly red rows).

BlocBuster compares the frequencies of each bloc between cases and controls, so it was necessary to correct for multiple testing. We employed two approaches for this task, Bonferroni correction and permutations of phenotypic labels, and observed that the theoretical and empirical approaches yielded similar thresholds for this study.

Finally, the association of the bloc was confirmed in an independent dataset of 439 psoriatic cases and 728 controls with even higher significances than the original dataset. Replication in independent data addresses another concern in GWAS: population stratification. Population stratification arises when a subset of individuals in the sample share some common ancestry and are overrepresented in either the cases or controls. In these scenarios, alleles associated with their ancestry might appear to be associated with the phenotype. In order to avoid this issue, samples are commonly chosen with as much population homogeneity as possible. Note that bootstrapping trials may be useful to flag potential stratification as they empirically quantify the sensitivity of the results to the selections of the samples. High variability of the test statistic over bootstrapping trials might represent population stratification. More formally, various statistical methods that utilize reference panels have been applied to test for stratification. However, Campbell et al. [72] demonstrated that these measures might not be adequate and showed that neither structured associations nor genomic control was able to correct for a false positive association of the *LCT* gene with human height. This association is believed to be false positive as it has not been validated. In general, testing on independent data is valuable for addressing the possibility of population stratification within GWAS. In this study, bootstrapping trials yield a tight confidence interval. Furthermore, replication in independent data provides an even stronger association than what was found using the original data. Taken together, it is not likely that population stratification inflated the association of *ps_1* with psoriasis. These results indicate this is a bona fide genetic pattern that is strongly associated with psoriasis.

BlocBuster is fundamentally different from standard network approaches in several ways. First, it introduces an extension of the CCC metric [73]. CCC is unique in that it computes a multi-faceted collection of correlation values, thereby accommodating heterogeneity. This accommodation is continued through network construction by expanding the network scaffolding. Note that in general, the degree of heterogeneity and the number of identified interacting markers can oppose each other during searches for associations. As the number of identified markers is increased, the pattern becomes increasingly refined. On one hand, this specificity has more power to pinpoint individuals bearing an epistatic pattern that contributes to the phenotype. On the other hand, if this pattern partially overlaps with another associated epistatic pattern, inclusion of markers that are not common to both patterns can weaken associations with the phenotype. For this reason, heterogeneity should be integrally accommodated throughout the analysis, as is done by BlocBuster.

Second, the networks created by other methods typically are comprised of a single connected component that must be partitioned using some type of clustering strategy. It is not clear which clustering strategy is the most appropriate and selections of parameters, e.g. numbers of clusters, can introduce bias and/or error. In contrast, BlocBuster networks are comprised of discrete components that are not connected to one another, so there is no ambiguity about how to separate them. Third, traditional approaches do not provide specific information regarding the genetic patterns that have variations between populations, while BlocBuster explicitly defines these allelic patterns as well as the relevant statistics. Finally, many other methods rely upon matrices comprised of similarities between pairs of individuals while BlocBuster is based upon correlations between SNP alleles, not individuals. On a related note, BlocBuster supplies a different type of information than the LD structure captured by tools such as Haploview [74], as it captures correlations that have potential to span any genomic distance while omitting uncorrelated markers, and it supplies the specific alleles that are correlated, not just the SNPs.

When compared with Pearson's correlation coefficient (PCC), CCC identified more than twice as many correlations for the 17 SNPs in *ps_1* and PCC split this bloc into pieces. More generally, PCC was more likely to introduce type I errors, and the PCC network had lower community structure than the corresponding CCC network, with the edges more dispersed throughout the network. As a practical consideration, PCC required three times as much computation time.

BlocBuster is a computationally efficient approach for GWAS. In general, direct identification of combinations of three or more inter-correlated markers is infeasible for GWAS due to the extremely large number of combinations. Given a pairwise correlation metric that can be quickly computed, network methods are efficient alternatives as they compute all pairwise correlations and build a network with potential to render higher-ordered inter-correlations. CCC is a straight-forward and simple metric and computations for the dataset of 1,610 individuals and 443,020 SNPs required a total of about 54 days. We ran the analysis on a cluster of 45 processors, which produced the results in less than a day and a half. At this rate, scaling up to 3,000,000 SNPs would require approximately 490 days of computation on a single CPU, which can be performed in parallel in a reasonable amount of time, given adequate resources (e.g. about five days on a cluster of 100 processors). Extraction of the blocs from the network using a breadth-first search and evaluating the associations of these blocs required only four minutes of computation time on a single processor.

BlocBuster expands upon the previous CCC implementation [73] in several key ways. First, the previous approach was unable to accommodate missing data. Note that imputation of missing data prior to computing correlations can generate false positive signals. Such imputations are based upon the assumption of LD between missing and identified markers [75]. While imputation can be useful for association studies in which each SNP is considered individually, errors introduced are biased toward inflated LD, which is a property captured by correlation measures. Consequently, the previous CCC implementation required elimination of all SNPs containing any missing data, which can lead to a significant loss of information. An advantage of requiring complete data is that there are only three possible states for each biallelic SNP genotype and the previous implementation leveraged this property with an encoding and table look-up method that increased computational efficiency. This approach is not practical for more than three states, thereby occluding extension to accommodate missing data or multi-allelic data. In view of these limitations, the CCC algorithm introduced for BlocBuster is an entirely different approach and employs a simple tallying method that is similar to Plink's Fast Epistasis implementation [31]. This substitution has yielded some loss of computational speed. However, BlocBuster's CCC is still three times faster than Pearson's correlation coefficient (PCC) and retention of SNPs with small percentages of missing values is a valuable property. Furthermore, BlocBuster can be readily extended to analyze multi-allelic data. It should be noted that missing data may yield unexpected results when combinations of SNPs are analyzed and BlocBuster incorporates several mechanisms to flag any potential issues (see Methods). A second key difference is that the previous CCC implementation built two separate networks for cases and controls and correlations were computed for each group separately. This strategy can facilitate identification of subtle patterns. However, spurious correlations arising in one group's data might not appear in the other group, subsequently confounding tests of statistical significance of case/control associations. Third, the previous CCC approach did not include any type of validation methods. While replication using independent datasets is ideal, suitable data are often expensive to obtain. This situation is especially problematic for endophenotypes, such as gene and protein expression, in which it is unlikely that another lab would have produced such data independently. It can also be an issue for well-studied phenotypes due to population-level variations across independent studies. BlocBuster provides a series of validation trials for efficient yet rigorous screening of results, thereby weeding out weak associations prior to further investments. In addition to these key differences, BlocBuster is a fully developed software package and the C++ source code is freely available. Using this package we have identified a novel combinatorial genetic pattern that is significantly associated with psoriasis, improving upon previous results by providing a genetic fingerprint of 17 SNP alleles that supplies specific information along a 211 kb span in the MHC region. This pattern was extensively computationally validated and confirmed in independent data.

Two caveats of our approach should be noted. First, BlocBuster is not designed to identify a marker that is independent in its association with a phenotype. There must be at least two correlated markers with a combination that exhibits association for recognition by this approach. For this reason, studies should employ standard single-marker association tests in addition to BlocBuster. Second, BlocBuster is diligent in removing false positives and consequently may miss true signals. However, this quality ensures the usefulness of the identified blocs for assessing risk and exploring pathogenesis.

GWAS are prevalent for phenotypes of scientific interest and future advances hinge on embracing the complexities of combinatorial interactions. Previous approaches for identifying combinatorial patterns presented biases as they relied upon known information and previous correlation measures were weakened by heterogeneity. BlocBuster's multi-faceted approach and allele-specific network efficiently accommodate heterogeneity. The benefits of this accommodation are highlighted by the comparisons with Pearson's correlation coefficient, which clearly demonstrate higher accuracy and reduced type I errors for our approach. Looking forward, BlocBuster's efficient selection of correlated markers will further increase the value of this approach as genetic data become increasingly affordable and dense.

## Materials and Methods

### Psoriasis datasets

The genotyping of samples was provided through the Genetic Association Information Network (GAIN). The datasets used for the analyses described in this manuscript were obtained from the GAIN Database at http://view.ncbi.nlm.nih.gov/dbgap-controlled through dbGaP accession number phs000019.v1.p1.c1, General Research Use (GRU) and Autoimmune Disease Only (ADO). Perlegen 500K chips were utilized for both studies.

The GRU dataset included 451,724 SNPs for 1,683 individuals. The removal of mitochondrial DNA and the X and Y chromosome SNPs resulted with 443,020 autosomal SNPs. We removed the children as well as the individuals that did not pass quality control (defined by the original study). We checked this extracted data for percentages of missing values and found that each of the 1,610 individuals and each of the 443,020 SNPs had no more than 10% missing data. In total, 929 and 681 of these individuals were labeled as psoriatic cases and normal controls, respectively.

The ADO data consisted of 451,724 genotypes for each of 1,214 individuals. We removed the individuals that did not pass previously defined QC, leaving 1,167 individuals. We removed SNPs from the mitochondrial DNA and X and Y chromosomes, leaving 443,020 SNPs. Three of these SNPs possessed between 10% and 20% missing data and the rest had less than 10% missing values. None of the 1,167 individuals had more than 10% missing data. The final dataset included 439 cases and 728 controls.

### BlocBuster

#### CCC

Our open-source code is available at www.blocbuster.org or by contacting the first author. BlocBuster is a network model that employs a custom correlation coefficient (CCC) [73]. High efficiency is achieved by computing a simple calculation that returns a vector of four values for each SNP pair. Briefly, let *A* and *a* represent the two alleles at the first SNP, and *B* and *b* represent the two alleles at the second SNP. There are four allelic combinations defined by these two SNPs: *AB*, *Ab*, *aB*, and *ab*. Each of these four combinations is evaluated independently of each other, providing a vector of four values that are utilized for network construction as described below.

Note that missing data should not be imputed prior to computing correlations as imputation errors are biased toward inflated LD. On the other hand, the retention of SNPs with missing data can yield unexpected consequences when combinations of SNPs are analyzed. For example, two SNPs with 20% missing data could results with up to 40% of the individuals missing one genotype value, and these individuals should not be included in an assessment of correlation between the two SNPs. Furthermore, when assessing associations for a combination of SNP alleles, the inclusion of individuals with excessive missing data for the combination can yield misleading results. BlocBuster employs several mechanisms to flag potential issues that might arise. BlocBuster omits individuals with missing genotypes for either of the SNPs in the given pair when computing CCC and warnings are printed out whenever a pair of SNPs possesses more than a user-defined percentage of individuals with missing data. Furthermore, each bloc identified as exhibiting a significant association (see Statistical Analyses below) has the actual sample sizes utilized in the analysis printed out. Finally, all individuals are included in the visual inspection of the bloc (to be described shortly), with the missing genotypes represented by white cells. These mechanisms ensure that each pattern is correctly represented without imposing risks of imputation errors contributing to misleading associations.

#### Network construction

**Figure 6:**
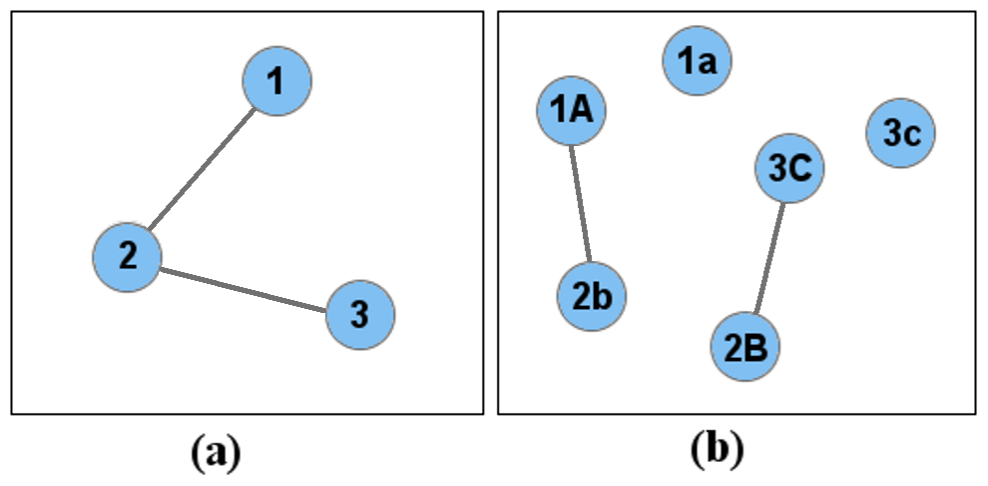
Comparison of SNP network construction methods. The network in (a) represents each SNP by a single node, as is done by previous approaches, and the network in (b) employs a node for each of the SNP alleles. Allele ‘A’ for SNP 1 is correlated with allele ‘b’ of SNP 2, and allele ‘B’ of SNP 2 is correlated with allele ‘C’ of SNP 3. Allelic information is lost in a standard network (a), resulting with an incorrect linking of allele 1A with 3C. The BlocBuster approach also enables allele-specific testing of bloc patterns.

Standard SNP networks consist of *n* nodes, where *n* is equal to the number of SNPs. Consequently, the allele states that exhibit correlations are lost in the network abstraction (Figure 6). To overcome this loss of information, each BlocBuster network is constructed with 2*n* nodes and each SNP is represented by two nodes, one for each of its alleles (the current implementation assumes biallelic SNPs). This strategy supplies the actual alleles contributing to a pattern, not just the SNPs, and enables allele-specific testing of patterns. It may also reduce erroneous merging of distinct patterns into a single bloc, as shown in Figure 6.

CCC is computed for each pair of SNPs, providing a vector of four values for each computation, with each value representing each of the possible SNP allele combinations, as described above. Then the *n* highest CCC values are represented by edges in the network. Note that it is possible for one pair of SNPs to have more than one edge in the network and it is not uncommon to observe a high correlation between an allele pair as well as mirrored correlation between their alternate alleles. On the other hand, mirrored correlations may not exist, as demonstrated in Figure 1. The two nodes representing the SNP alleles for each high correlation are connected with an edge. The resultant network is comprised of 2*n* nodes and *n* edges, representing the *n* highest CCC values over all pairs of SNPs, resulting with an average node degree of one. If the edges were uniformly distributed, the network would consist of 443,020 *doubletons*, each of which was comprised of a single edge connecting two nodes; with every node connected to precisely one other node in the network. The choice of this number of edges is somewhat arbitrary, but any smaller number of edges would make it impossible for every node in the network to be connected to at least one other node, and a larger number of edges would result with a less stringent CCC threshold. The second set of permutation trials indicated that the network density could be substantially increased with low risk of introducing false-positive edges, so the number of edges could be increased. However, increasing the number of edges yields non-decreasing bloc size and non-increasing frequencies of the bloc pattern. In essence, the use of a stringent CCC threshold captures only the strongest of correlations, thereby facilitating the discovery of strong genetic patterns within the data.

#### Bloc extraction

We have observed that BlocBuster networks built from biological data consist of large numbers of singleton nodes (with no edges) and discrete blocs of nodes with relatively high densities of edges. Consequently, no clustering methods or other types of partitioning are required to separate the network. It is straight-forward to deterministically extract the blocs with a breadth-first search of the network. The SNP allele pattern that corresponds to each bloc is then tested for associations between two sets of individuals, with those that exhibit significant associations being reported to the user.

#### Software

Our open source software is freely available at www.blocbuster.org or by contacting the first author. The code was written in C++ and implemented and tested on a LINUX platform.

### Statistical Analyses

#### G-test and odds ratio

An underlying entailment of most existing network approaches is that correlations are transitive. For example, when grouping markers into clusters, where for three markers, *A*, *B* and *C*, in one cluster, if *A* is correlated with *B* and *B* is correlated with *C*, then *A* is implied to be correlated with *C*. However, this hidden assumption might not be valid, or even problematic, as different subsets of individuals may be contributing to each of the correlations. Consider the case where individuals bearing both *A* and *B* or bearing both *B* and *C* are at high risk for the disease, but individuals with only *A* and *C* are not at increased risk. Such a case would violate transitivity. For this reason, BlocBuster tests each of these patterns by tallying the frequencies of the bloc in its entirety. Furthermore, because we are interested in identifying associations with complex phenotypes, we compare the frequencies between phenotypic groups. Such tests are not straightforward for existing network approaches as current correlation metrics only indicate which pairs of SNPs are correlated and do not supply which alleles of the SNP pairs contribute to the correlations.

Since the networks were blindly built using a combination of the data for all of the individuals, it could be expected that most blocs are common at the population level, with no association to the phenotype. However, because CCC accommodates genetic heterogeneity, it might also be expected that some of these blocs exhibit significant association with the phenotype status, which is one type of heterogeneity. Three metrics were used to evaluate the significance of these patterns: odds ratio, Bonferroni-corrected p-value based on the G-test of independence, and corrected p-value based on permutation trials. These metrics were computed for all blocs that were possessed by at least ten individuals in the entire sample. Individuals that have more than 5% missing genotype values for the SNPs in a given bloc were not included in the computations. However, these individuals were included in the plots of genotype values for visual inspections, as described below.

The odds ratio was computed as *OR* = *ad*/*bc*, where *a* and *b* equal the numbers of the bloc of interest possessed by the cases and controls, respectively. Each individual possesses two allelic combinations that include the SNPs in the bloc. The sum of the combinations that are not identical to the given bloc is represented by *c* for the cases and by *d* for the controls. (Stated in another way, the odds ratio is equal to *p*(1-*q*)/*q*(1-*p*), where *p* and *q* equal the frequencies of the bloc for cases and controls, respectively.) The 95% confidence interval is defined as e ∧ [*ln*(OR)+/−1.96 *sqrt*(1/*a*+1/*b*+1/*c*+1/*d*)], where *ln*(OR) equals the natural log of *ad*/*bc* and *sqrt* is the square root.

G-test is a maximum likelihood statistical significance test which converges to the chi-squared distribution more accurately than Pearson's chi-squared test [76]. The p-value of significance corresponding to the G-test score, with Bonferroni correction for multiple testing, was used for evaluating each bloc. We used the following formula for the G-test of independence:
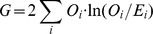
where *O_i_* and *E_i_* equal the observed and expected number of entire bloc patterns in subset *i*. The expected numbers of blocs were computed using the observed numbers of blocs as a 2×2 table, where the null hypothesis is that the relative proportions of each variable are independent of each other. The summation is over four subsets (corresponding to the four cells of the 2×2 table), and ‘ln’ denotes the natural logarithm.

#### Permutation trials for multiple testing corrections

In addition to using Bonferroni correction, an empirical approach via permutation trials was employed. We permuted the phenotype labels across the samples, tallied the numbers of carriers of each bloc for each of these randomized groups of individuals, and computed the G-test scores. There were 1,000 permutations and all 54,425 blocs in the original network were tested for each permutation. For each permutation, the individuals were randomly assigned into groups of 929 and 681 individuals and the bloc frequencies in these two groups were compared.

### Validation

In order to ensure the robustness of our results, we employed a series of validation trials, as follows.

#### Permutation trials for assessing type I errors

Permutation trials were utilized to test whether significant correlations might have arisen in the data by random chance. In these trials the genotypes of the individuals were randomly shuffled for each SNP. Consequently, each SNP had its original allele and genotype frequencies; the only change was that the inherent “correlations” between pairs of SNPs had been removed by randomly rearranging the genotypes across individuals. After each randomization, the CCC values for every pair of SNPs were computed. These trials indicated the likelihood that type I errors arose during computations of correlations; such errors would create false-positive edges in the network.

#### Variation of network density

To test the robustness of the blocs to variation of network density, we built a series of networks with *n* edges representing the *n* highest CCC values. We varied *n* from 50,000 to 500,000 edges, at 50,000 increments, and tracked the bloc of interest through these varying densities.

#### Bootstrapping trials

Bootstrapping trials were employed in which we randomly selected half of the cases and half of the controls and computed the odds ratios and p-values for the bloc of interest. These randomized trials were repeated 1,000 times, with the odds ratio and p-value recorded for each trial. The means and the 95% confidence intervals over the 1,000 trials for each of these statistics were computed.

#### Visual inspection

The genotype values for the significant bloc were plotted for the psoriatic cases and controls for visual inspection. We utilized our previously developed rearrangement clustering method, TSP+*k* [77], to reorder the individuals (rows) and place similar individuals near each other. Briefly, the genotype values for the SNPs for each bloc were extracted from the data and converted to an instance of the Traveling Salesman Problem (TSP) [78] in which each individual was represented as a city. We inserted three dummy cities to facilitate three clusters and solved the optimal ordering of the cities using NEOS's [79] Concorde solver (www.tsp.gatech.edu/concorde.html). The individuals were reordered using this solution. Using this ordering of individuals, the genotypes were color-encoded with dark blue, light blue, red, and white representing homozygote for the protective allele, heterozygote, homozygote for the risk allele, and missing data, respectively. The matrices of genotypic values were plotted for the cases and controls, as shown in Figure 3.

#### Replication in independent data

We tested the identified psoriasis bloc for phenotypic association in an independent data set, the Genetic Association Information Network (GAIN) Autoimmune Disease Only (ADO) data. These data are described at the beginning of this section.

## Supporting Information

Table S1 **List of SNP alleles in 30-node bloc from 500,000 edge network.**
(DOC)
