# Supplementary material for: Allele-Specific Network Reveals Combinatorial Interaction That Transcends Small Effects in Psoriasis GWAS
Source: PLoS Comput Biol. 2014 Sep 18;10(9):e1003766. doi: 10.1371/journal.pcbi.1003766 (PMC4168982; doi:10.1371/journal.pcbi.1003766)
Supplement: Table S1 — List of SNP alleles in 30-node bloc from 500,000 edge network. (DOC) [file pcbi.1003766.s001.doc]

**Allele-specific network reveals combinatorial interaction that transcends small effects in psoriasis GWAS**

Sharlee Climer^a,*^, Alan R. Templeton^b,c,d^, Weixiong Zhang^a,c,e*^

Departments of ^a^Computer Science and Engineering, ^b^Biology, ^c^Genetics, Washington University, St. Louis, MO 63130, USA

^d^Institute of Evolution, and Department of Evolutionary and Environmental Biology, University of Haifa, Haifa 31905, Israel

^e^Institute for Systems Biology, Jianghan University, Wuhan, Hubei 430056, China

*Correspondence: E-mail: [climer@wustl.edu](mailto:climer@wustl.edu) or [weixiong.zhang@wustl.edu](mailto:weixiong.zhang@wustl.edu).

SUPPLEMENTARY INFORMATION

Contents Page

**Table S1.** List of SNP alleles in 30-node bloc from 500,000 edge network …………...………………… 2

**Table S1**  List of 30 SNP alleles in the expanded bloc that appeared in the 500,000 edge BlocBuster network. Seventeen of these SNPs comprise *ps_1*.

| **ss_ID** | **PGP_ID** | **Protective Allele** | **Freq. Cases** | **Freq. Controls** |
| --- | --- | --- | --- | --- |
| ss68971177 | PGP06300240 | A | 0.569 | 0.676 |
| ss68971246 | PGP12151862 | C | 0.606 | 0.734 |
| ss68971259 | PGP04061061 | G | 0.619 | 0.748 |
| ss68971261 | PGP04396987 | C | 0.470 | 0.562 |
| ss68971276 | PGP07075713 | C | 0.596 | 0.744 |
| ss68971303 | PGP06710084 | C | 0.576 | 0.712 |
| ss68971335 | PGP06321811 | C | 0.702 | 0.850 |
| ss68971698 | PGP04062092 | A | 0.817 | 0.831 |
| ss68971708 | PGP00291692 | A | 0.818 | 0.831 |
| ss68971723 | PGP06807680 | A | 0.820 | 0.832 |
| ss68971871 | PGP00292092 | A | 0.714 | 0.802 |
| ss68971880 | PGP08019425 | A | 0.705 | 0.782 |
| ss68971912 | PGP04056817 | A | 0.570 | 0.634 |
| ss68971109 | PGP05119742 | C | 0.531 | 0.654 |
| ss68971168 | PGP12151859 | G | 0.720 | 0.846 |
| ss68971195 | PGP00290874 | G | 0.579 | 0.700 |
| ss68971245 | PGP07353227 | G | 0.484 | 0.587 |
| ss68971249 | PGP04396979 | T | 0.609 | 0.740 |
| ss68971262 | PGP04061066 | T | 0.640 | 0.767 |
| ss68971345 | PGP07953531 | T | 0.487 | 0.599 |
| ss68971350 | PGP06770584 | G | 0.487 | 0.595 |
| ss68971355 | PGP05142391 | T | 0.659 | 0.792 |
| ss68971356 | PGP08276562 | G | 0.596 | 0.709 |
| ss68971359 | PGP05304455 | T | 0.704 | 0.846 |
| ss68971460 | PGP07775222 | G | 0.743 | 0.800 |
| ss68971717 | PGP07086629 | T | 0.666 | 0.751 |
| ss68971874 | PGP05521282 | G | 0.741 | 0.833 |
| ss68971883 | PGP07328342 | G | 0.810 | 0.814 |
| ss68971910 | PGP04056815 | T | 0.607 | 0.661 |
| ss68971911 | PGP04056816 | T | 0.571 | 0.621 |
